# A Novel Yolk–Shell Fe_3_O_4_@ Mesoporous Carbon Nanoparticle as an Effective Tumor-Targeting Nanocarrier for Improvement of Chemotherapy and Photothermal Therapy

**DOI:** 10.3390/ijms23031623

**Published:** 2022-01-30

**Authors:** Haina Tian, Ruifeng Zhang, Jiaqi Li, Cailin Huang, Wen Sun, Zhenqing Hou, Peiyuan Wang

**Affiliations:** 1 Research Center of Biomedical Engineering of Xiamen, Key Laboratory of Biomedical Engineering of Fujian Province, Department of Biomaterials, College of Materials, Xiamen University, Xiamen 361005, China; hainatian@163.com; 2 Key Laboratory of Design and Assembly of Functional Nanostructures, Fujian Institute of Research on the Structure of Matter, Chinese Academy of Sciences, Fuzhou 350002, China; zhangruifeng@mail.ustc.edu.cn (R.Z.); lijq7@mail.ustc.edu.cn (J.L.); Hhhayu@163.com (C.H.); 3 School of Rare Earths, University of Science and Technology of China, Hefei 230026, China; 4 State Key Laboratory of Fine Chemicals, Dalian University of Technology, 2 Linggong Road, Hi-Tech Zone, Dalian 116024, China; sunwen@dlut.edu.cn

**Keywords:** Fe_3_O_4_, mesoporous carbon, yolk–shell, MRI, chemotherapy, photothermal therapy

## Abstract

Owing to their good stability and high photothermal conversion efficiency, the development of carbon-based nanoparticles has been intensively investigated, while the limitation of unsatisfactory cellular internalization impedes their further clinical application. Herein, we report a novel strategy for fabrication of Fe_3_O_4_ yolk–shell mesoporous carbon nanocarriers (Fe_3_O_4_@hmC) with monodispersity and uniform size, which presented significantly higher cell membrane adsorption and cellular uptake properties in comparison with common solid silica-supported mesoporous carbon nanoparticles with core–shell structure. Moreover, the MRI performance of this novel Fe-based nanoparticle could facilitate precise tumor diagnosis. More importantly, after DOX loading (Fe_3_O_4_@hmC-DOX), owing to synergistic effect of chemo–phototherapy, this therapeutic agent exhibited predominant tumor cell ablation capability under 808 nm NIR laser irradiation, both in vitro and in vivo. Our work has laid a solid foundation for therapeutics with hollowed carbon shell for solid tumor diagnosis and therapy in clinical trials.

## 1. Introduction

Carbon-based nanomaterials, such as carbon nanotubes [1], graphene [2] and carbon nanohorns [3], has been extensively explored as drug delivery platforms for tumor therapy due to the ideal biocompatibility, unique photothermal conversion efficiency and other physiochemical properties [4,5,6,7,8,9,10]. In terms of nanostructural features, mesoporous carbon nanoparticles (MCN) are optimal for drug delivery, due to their well-defined mesoporous channels, large surface area and carbonaceous composition, endowing themselves with superior natural performance in comparison with mesoporous silica nanoparticles and other carbon nanomaterials [11,12,13,14,15]. However, specific surface area plays a vital role in drug delivery [16,17], tumor cell targeting and tumor retention [18,19]. Therefore, efforts have been made to optimize the porous parameters of mesoporous nanoparticles, especially the pore volume, pore size and more importantly, the specific surface area, in order to achieve the goal of improving the loading capacity of the therapeutic cargo [20,21]. Fortunately, the introduction of a novel hollow cavity into the core of mesoporous nanoparticles is an optimally effective way to remarkably increase the pore volume relative to the weight or specific surface area [22,23]. Moreover, it is capable of tuning and regulating the hollow cavity area and the thickness of the mesoporous shell. This innovative structure is of critical concern for targeting delivery platforms, because massive drug molecules could be encapsulated inside of this large hollow cavity, rather than only loosely adsorbed on pore walls, resulting in sustaining leakage in normal tissue [24,25]. Undoubtedly, it is a meaningful challenge to develop a facile strategy to construct a well-controlled, carbon-based nanoparticle with virus-inspired surface and open mesoporous channels, so as to reach the requirement of effective cellular internalization in different biomedical applications.

Recently, magnetic nanoparticles have received great attention owing to their unique bio-application, such as in the contrast agents of magnetic resonance imaging, magnetic targeting in tumor sites and as magnetic storage media [26,27,28,29,30,31]. As favorable biomedical nanoparticles, Fe_3_O_4_ (iron oxide) nanospheres have been intensively developed due to their unique features, such as low toxicity, superior biocompatibility, sensitive response toward magnetic field and facile fabrication with tunable sizes [32,33,34,35]. More importantly, the FDA has approved Fe_3_O_4_ as a contrast agent for T2-weighted MRI. Moreover, it has been widely explored in various biomedical applications, such as drug delivery targeting, magnetic heat administration and MRI [36]. Unfortunately, conventional Fe_3_O_4_ is often aggregated in the biological media and the specific surface area limitation substantially impedes their clinical translation as therapeutic agents. To solve the above dilemmas, surface passivation or surface coating of mesoporous silica are the most frequent strategies for enhancing the stability and specific surface area [37,38]. It is well known that mesoporous silica shells can be simply coated on various kinds of nanomaterials for the prevention of the aggregation phenomenon in biomedical applications and act as drug delivery platforms [39,40,41,42]. Based on the above results, exploration of further surface modification approaches for expanding iron oxide application in clinical applications is urgent.

**Figure 1:**
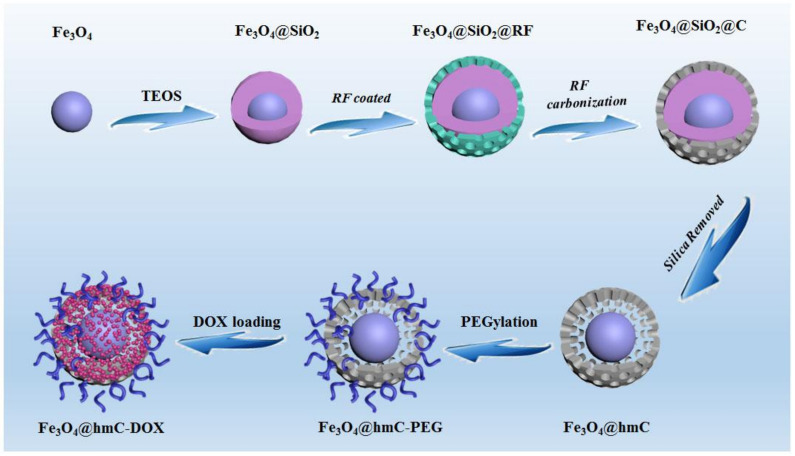
Fe_3_O_4_@hmC stepwise fabrication via hard template approach.

In this work, we report the facile synthesis of hollowed mesoporous carbon-coated Fe_3_O_4_ nanoparticles with a yolk–shell nanostructure (Fe_3_O_4_@hmc), which could be used as a drug delivery system and as photothermal agents against tumor cells. Owing to the outside layer of mesoporous resorcinol–formaldehyde spheres shells, according to the transmission electron microscopy (TEM) and scanning microscopy (SEM) images, the obtained Fe_3_O_4_@hmc has an unparalleled hollowed cavity and mesoporous structure (Figure 1), which were preferentially internalized by tumor cells in comparison with surface solid-supported mesoporous carbon nanoparticles with the same size. The photothermal conversion efficiency test demonstrated the carbon-based nanoshells have a superior light absorption capability. After DOX is encapsulated in the hollowed mesoporous carriers, numerous tumor cells are killed, ascribing to chemotherapy and photothermal therapy (PTT). Moreover, the core Fe_3_O_4_ nanocrystals can be used as MRI contrast agents, given that tumor outline was successfully delineated after tail vein injection of our yolk–shell nanocarriers. Moreover, solid tumors were effectively eliminated in breast tumor-bearing mice under 808 nm laser irradiation. These results proved that these hollowed mesoporous carbon-coated Fe_3_O_4_ nanoparticles have potential for application as optimal, excellent contrast agents and multi-mode therapeutics for chemotherapy and PTT.

## 2. Results and Discussion

### 2.1. Fe_3_O_4_@hmC Fabrication

**Figure 2:**
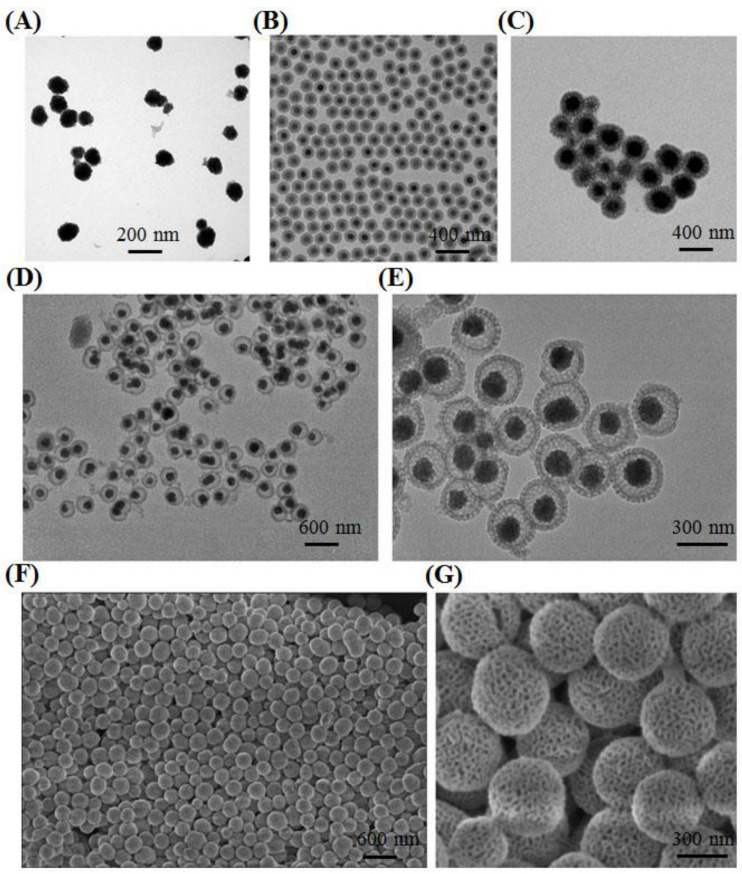
TEM images of Fe_3_O_4_ (**A**), Fe_3_O_4_@SiO_2_ (**B**), Fe_3_O_4_@SiO_2_@C (**C**); large scale (**D**) and magnified (**E**) TEM image yolk–shell Fe_3_O_4_@hmC. Large scale (**F**) and magnified (**G**) SEM image yolk–shell Fe_3_O_4_@hmC.

Fe_3_O_4_ nanocrystals with size of ~120 nm were obtained by a conventional hydrothermal method [43]. As shown in Figure 2A, the aggregation phenomena of as-prepared nanocrystals were found in aqueous solution. Hence, in order to resolve this limitation, solid silica was then applied to wrap on the surface of Fe_3_O_4_ (Fe_3_O_4_@SiO_2_). TEM images of as-fabricated core–shell nanoparticles proved that solid silica was successfully coated on the surface of iron oxide; meanwhile, the uniformed nanospheres presented superior dispersion in comparison with free iron oxide, attributing to the silicon hydroxyl in the solid silica surface (Figure 2B). Then, the mesoporous resorcinol and formaldehyde (RF) shell was coated on Fe_3_O_4_@SiO_2_, so that a unique abundance of radial and oriented mesoporous channels were constructed with Pluronic F127 as the common structural directing agent and 1,3,5-trimethylbenzene (TMB) as the mediator in the water and ethanol mixture system. As displayed in Figure S1, the second layer can be clearly observed and distinct mesoporous RF were homogenously dispersed on the surface (Fe_3_O_4_@SiO_2_@RF). Typically, as a polymer with highly cross-linked performance, RF can be successfully converted into carbon with high yield, making them optimal candidates in various carbon-related nanomaterials fabrication. Therefore, after carbonization under the protection of nitrogen gas at 800 °C, the RF shell could be successfully transformed into carbon (Fe_3_O_4_@SiO_2_@C) (Figure 2C). In order to obtain a high specific surface area, the solid silica layer was finally etched by 0.1 M NaOH under 60 °C; the last product, Fe_3_O_4_@hmC with a yolk–shell nanostructure and a carbon-based, well-ordered mesoporous channel surface, was successfully prepared (Figure 2D,E). This mesoporous carbon shell also exhibited radially oriented mesopores that launched out from the center to the shell. Compared with conventional, honeycomb-like mesoporous structures, these unique, divergent mesoporous channels could facilitate the NIR light directly, without any substrate blocking. The surface morphology and mesopores of Fe_3_O_4_@hmC were then evaluated by scanning electron microscopy (SEM). As shown in Figure 2F,G, uniformed Fe_3_O_4_@hmC could be found under SEM and distinct various pore distribution on the carbon shell could be clearly observed, which were consistent with the TEM images.

### 2.2. Fe_3_O_4_@hmC Characterization

**Figure 3:**
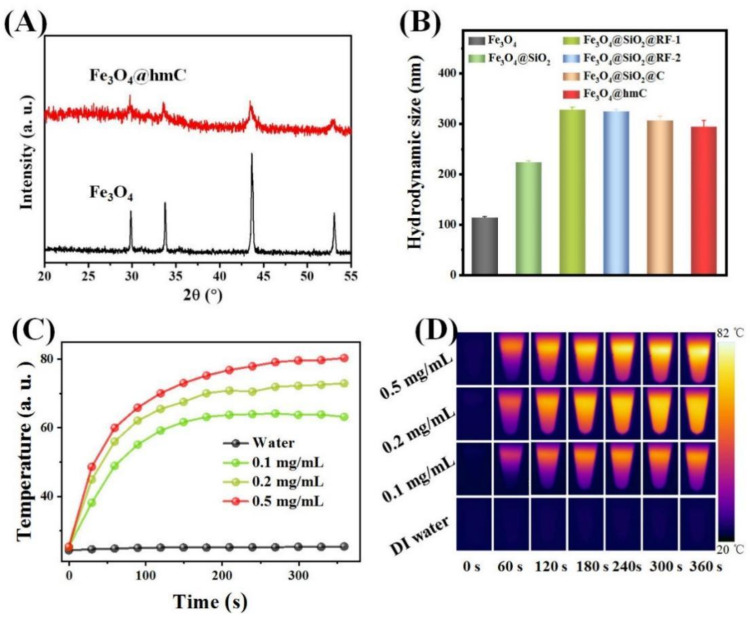
XRD patterns of Fe_3_O_4_@hmC and Fe_3_O_4_ (**A**). Average size of Fe_3_O_4_, Fe_3_O_4_@SiO_2_, two kinds of Fe_3_O_4_@SiO_2_@RF, Fe_3_O_4_@SiO_2_@C and Fe_3_O_4_@hmC (**B**). NIR induced heat generation of Fe_3_O_4_@hmC with different concentrations under 808 nm laser irradiation, 0.9% NaCl was set as the control (**C**). The photothermal images of Fe_3_O_4_@hmC with different concentrations and 0.9% NaCl after 808 nm laser irradiation for various times (**D**).

**Figure 4:**
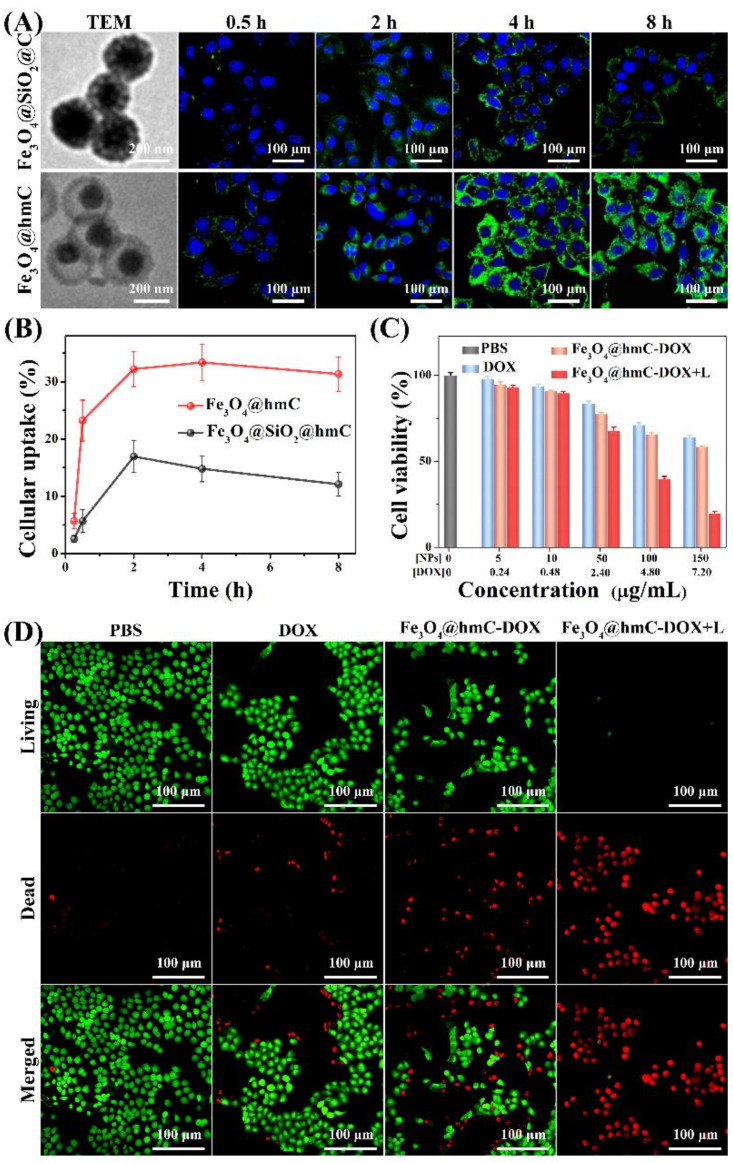
TEM images and CLSM images of Fe_3_O_4_@ SiO_2_@C with core–shell structure, Fe_3_O_4_@hmC with yolk–shell structure after incubated with 4T1 cells for various durations (**A**). Cellular uptake percentage of Fe_3_O_4_@ SiO_2_@C and Fe_3_O_4_@hmC after being co-cultured with 4T1 cells for different durations (**B**). Cell viability (**C**) and fluorescence images (**D**) of live/dead cells after different treatments of PBS, DOX, Fe_3_O_4_@hmC-DOX and Fe_3_O_4_@hmC-DOX with 808 nm laser.

After yolk–shell nanospheres fabrication, in order to investigate the presentation of the Fe_3_O_4_ core, X-ray diffraction (XRD) patterns of Fe_3_O_4_@hmC and ligand-free Fe_3_O_4_ were subsequently studied. All of the distinct diffraction peaks in Fe_3_O_4_@hmC were exclusively attributed to Fe_3_O_4_ (No. 19-0629, JCPDS) without any other phase impurity, demonstrating that no reduction of Fe^3+^ to Fe^2+^ occurred during the carbonization process under high temperature (Figure 3A). The attenuated characteristic peaks could be ascribed to the amorphous station of the carbon shell. Interestingly, we found that, after 2 shells were coated, the average size of Fe_3_O_4_@SiO_2_@RF gradually increased from 125 nm (Fe_3_O_4_) to 324 nm; meanwhile, after calcination, Fe_3_O_4_@SiO_2_@C size shrunk to 306 nm (Figure 3D). Accordingly, pure RF resin was fabricated via the literature reported, with water as the solvent [44], while the heating of the RF shell resin in the nitrogen atmosphere to 750 °C could lead to weight loss of the adsorbed water molecular which induced the decrease in size. Recently, carbon-based, near-infrared region (NIR) resonant nanomaterials, such as carbon nanotubes, graphene oxide and carbon nanohorns, have been extensively explored for hyperthermia cancer treatment [1,2,3,4,5,6,7,8,9,10]. These nanomaterials have an ideal photothermal conversion capability which could convert the NIR light into heat to eradicate cancerous cells. Owing to the high NIR absorption of this carbon-based nanocomponent (Figure S2), we then evaluated the hyperthermia induction efficiency of our Fe_3_O_4_@hmC under 808 nm laser exposure through an NIR thermal camera. The yolk–shell nanomaterials were dispersed in aqueous solution (0.9% NaCl) at three different concentrations (0.1 mg/mL, 0.2 mg/mL, and 0.5 mg/mL) with 808 nm laser irradiation, and then the temperature of the as-prepared samples was recorded. As shown in Figure 4, the heat rate of Fe_3_O_4_@hmC increased as the function of concentration increased from 0.1 to 0.5 mg/mL, proving that NIR-induced hyperthermia growing presented a concentration-depended behavior. More importantly, after only 6 min illumination, the temperature could reach 80 °C, while the temperature has negligible fluctuation in 0.9% NaCl group, further demonstrating the superior heat conversion capability of Fe_3_O_4_@hmC for photothermal therapy application.

### 2.3. In Vitro Photothermal Tumor Cell Killing Ability of Fe_3_O_4_@hmC

Encouraged by the unique adsorption capacity property of our yolk–shell Fe_3_O_4_@hmC toward cytomembrane, cellular internalization capability was then evaluated in a 4T1 cell line. In order to investigate the advantage of cellular internalization toward tumor cells, conventional, mesoporous, carbon-coated Fe_3_O_4_ core–shell nanoparticles with no cavity space (Fe_3_O_4_@SiO_2_@C) were set as the control (Figure 4A). Fe_3_O_4_@SiO_2_@C with the size of 306 nm was finally loaded with the same FITC dose as Fe_3_O_4_@hmC with the size of 294 nm (Figure S3). Confocal laser scanning microscope (CLSM) was used to evaluate the endocytosis efficiency of the above two nanoparticles. As shown in Figure S4 and Figure 4, green fluorescence could be detected only after 15 min incubation in Fe_3_O_4_@hmC, while unobvious signals were observed in the Fe_3_O_4_@ SiO_2_@C group; after 0.5 h co-culture, both of the carbon-based nanocomponents could be clearly found in the cytoplasm of 4T1 breast tumor cells. Cellular internalization of Fe_3_O_4_@hmC has time-dependent behavior after 2 h co-culture, with intracellular green signals becoming the highest, and a slight decrease after 4 h incubation. Importantly, Fe_3_O_4_@hmC presented significantly stronger signals than Fe_3_O_4_@ SiO_2_@C, especially at 2 h incubation, demonstrating that the corresponding large cavity of hollowed shell can effectively adsorb on the cytomembrane and accumulate in the whole cytoplasm, indicating that hollowed structure nanoparticles were the ideal drug delivery nanoplatforms (Figure 4A). Besides, after quantitative analysis of the cellular uptake percentage via inductively coupled plasma mass spectrometry (Fe^3+^ caculation), Fe_3_O_4_@hmC presented remarkably higher endocytosis in comparison with Fe_3_O_4_@ SiO_2_@C in each time point of incubation. Meanwhile, Fe_3_O_4_@hmC exhibited longer intracellular retention after 8 h incubation, which can be ascribed to the high adsorption capacity of hollowed mesoporous nanoparticles toward organelles, implying the long tumor retention capability of our yolk–shell nanoparticles with large cavity space (Figure 4B). The carbonaceous shell can interact with DOX molecules via π-stacking or hydrophobic–hydrophobic interactions, which could be easily triggered to release under an external acid microenvironment [45]. Therefore, the cavity of our Fe_3_O_4_@hmC was loaded with DOX (Fe_3_O_4_@hmC-DOX) for combination eradication of tumor cells via chemotherapy and photothermal therapy. Firstly, 4T1 cell killing efficiency was estimated by MTT strategy. Interestingly, Fe_3_O_4_@hmC-DOX exhibited lower cell viability, suggesting higher cytoplasm DOX delivery in comparison with free DOX. Obviously, after laser irradiation, the cell-killing percentage of Fe_3_O_4_@hmC-DOX was predominantly higher than other groups, thus confirming that carbon shell possessed the ability to absorb NIR laser and induced cytotoxicity by heat. Furthermore, live/dead cell kit, Calcine-AM/PI, was also used to explore the exact cell killing efficiency after PBS, DOX, Fe_3_O_4_@hmC-DOX and Fe_3_O_4_@hmC-DOX with laser treatment. Red signals of dead cells in the Fe_3_O_4_@hmC-DOX+laser group presented significant cell apoptosis, whereas free DOX and Fe_3_O_4_@hmC-DOX can only induce partial 4T1 cells death, further demonstrating that heat triggered by the carbon shell under NIR light and free DOX, gradually released from Fe_3_O_4_@hmC-DOX, could effectively trigger tumor cells damage.

### 2.4. MRI and Photothermal Imaging

**Figure 5:**
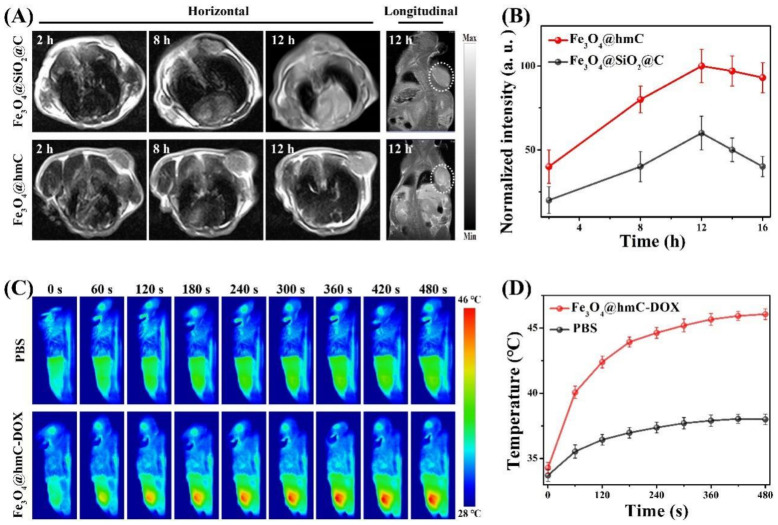
Horizontal and longitudinal MRI results of breast tumor bearing mice after tail vein injection of Fe_3_O_4_@hmC or Fe_3_O_4_@SiO_2_@C for different hours (**A**). Quantitative analysis of MRI signals after tail vein injection of Fe_3_O_4_@hmC or Fe_3_O_4_@SiO_2_@C for different hours (**B**). Photothermal images (**C**) and temperature curve (**D**) of breast tumor bearing mice after 12 h post-injection of Fe_3_O_4_@hmC-DOX or PBS under 808 nm laser irradiation for various durations.

Inspired by the above superior cellular internalization investigation of our nanoplatform with yolk–shell morphology, we then carried out in vivo magnetic tumor-targeting evaluation of Fe_3_O_4_@hmC and Fe_3_O_4_@SiO_2_@C (same dose of Fe content), which were intravenously injected into 4T1 breast cancer-bearing mice. As shown in the T_2_-weighted MRI with the 5.0 T system, the T_2_-weighted signal of Fe_3_O_4_@SiO_2_@C and Fe_3_O_4_@hmC were gradually increased with maximum accumulation at 12 h post-injection. Importantly, tumor site longitudinal signals in Fe_3_O_4_@hmC were stronger with a more distinct tumor outline in comparison with Fe_3_O_4_@SiO_2_@C after 12 h injection (Figure 5A). Predominantly, quantitative MRI signals analysis of yolk–shell nanoparticles in the tumor tissue also exhibited higher results than those of core–shell Fe_3_O_4_@SiO_2_@C in every time point of injection, demonstrating that therapeutic agents with hollowed mesoporous structure could clearly promote the tumor accumulation and prolong the tumor retention (Figure 5B). Subsequently, we performed the photothermal conversion effect of our yolk–shell nanoparticles after tail vein injection of Fe_3_O_4_@hmC at 12 h via an infrared region thermal camera under 808 nm laser illumination. The temperature in the tumor site was raised to ~46 °C within 6 min in the magnetic group. In contrast, only ~38 °C increase was observed in the PBS group, further proving the photothermal conversion efficiency of carbon-based shells. These imaging results prove that Fe_3_O_4_@hmC-DOX is beneficial to the effective tumor-targeting capability for the combination of chemotherapy and photothermal therapy.

### 2.5. In Vivo Anti-Tumor Evaluation

**Figure 6:**
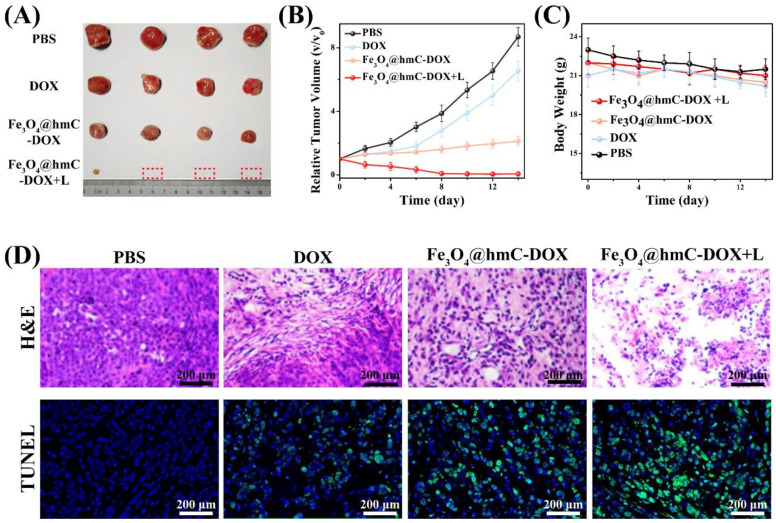
Photographs (**A**), tumor volume curve (**B**), body weight fluctuation (**C**) and histological staining (H&E and TUNEL) (**D**) of tumors after PBS, DOX, Fe_3_O_4_@hmC–DOX and Fe_3_O_4_@hmC-DOX+laser for 14 days; 808 nm laser was performed after 12 h injection of P Fe_3_O_4_@hmC-DOX. The dash box in A repents a total eradicated tumor.

Motivated by the effective tumor-targeting property and hyperthermia induction under an external NIR laser exposure, in vivo chemotherapy and photothermal ablation of breast solid tumor were performed. Mice with subcutaneous tumor were assigned randomly into four groups and received intravenous injection of PBS, DOX, Fe_3_O_4_@hmC–DOX and Fe_3_O_4_@hmC-DOX+laser. The corresponding laser irradiation was carried out after 12 h injection of Fe-based therapeutics. As shown in Figure 6A, after tumor resected at 14 days, only 1 tumor residual was detected in the Fe_3_O_4_@hmC–DOX+laser group, while Fe_3_O_4_@hmC–DOX and DOX only presented some extent of size reduction in comparison with the PBS group. Relative tumor volume curve exhibited that, in the chemotherapy and phototherapy combination group, tumors were substantially eradicated after 14 days of treatment. Some extent of tumor growth in free DOX and Fe_3_O_4_@hmC–DOX was perhaps due to the low passive targeting ability in DOX and the unsatisfactory chemotherapy efficiency in the nanocarrier (Figure 6B). Furthermore, tumor ablation of the solid tumor was estimated by histological examination and immunohistochemistry. The images from H&E analysis showed that tumor in the Fe_3_O_4_@hmC–DOX+laser group had extensive regions filling of shrinkage and fragmentation of apoptotic tumor cells and more quantity of abnormal cells were observed in this group in comparison with the other three groups. The TUNEL also presented some results that Fe_3_O_4_@hmC–DOX+laser group had the strongest green fluorescence signals of cell apoptosis/necrosis compared with the other three groups (Figure 6D). All anti-tumor results proved that our novel Fe_3_O_4_@hmC-DOX provided high therapeutic efficacy with the optimal synergistic effect of chemotherapy and photothermal therapy. Meanwhile, no body weight fluctuation and organs lesion could be detected in any of the four groups (Figure 6C and Figure S5), further indicating the low side effect of our ferrous and carbon-based nanoplatform—demonstrating the potential of clinical translation of this therapeutic agent.

## 3. Materials and Methods

### 3.1. Fe_3_O_4_ @hmC Fabrication

#### 3.1.1. Fe_3_O_4_ Nanoparticles Synthesis

The core Fe_3_O_4_ particles with hydrophilic property were firstly synthesized via a hydrothermal approach. Briefly, Fe precursor, 3.25 g FeCl_3_•6H_2_O, 1.3 g trisodium citrate and 6.0 g NaAc (sodium acetate) were mixed and dissolved in 100 mL ethylene glycol with vigorous magnetic stirring. Then, the above obtained yellow solution was carefully transferred into a 200 mL stainless steel Teflon-lined autoclave. The autoclave was sealed and heated for 200 °C for 10 h, and then the autoclave was cooled down under room temperature. Finally, the black precipitate was washed by deionized water 3 times and ethanol 3 times. The as-prepared Fe_3_O_4_ was re-dispersed in DI water.

#### 3.1.2. Fe_3_O_4_ @ SiO_2_ Fabrication

Then, the solid silica coating was realized as follows: 70 mL above-fabricated magnetite particles (0.02 g/mL) dispersed in DI water was firstly added to a round bottom flask with 3 necks. Then, 280 mL absolute ethanol and 5.0 mL stronger ammonia solution (28 wt%) were added to the above solution under gentle mechanical stirring at 30 °C for 15 min. Afterward, total 4.0 mL of TEOS was drop-wisely added within 2 min, and the above reaction was proceeded for 8 h under continuous stirring. The mole ratio of all reagents, TEOS: ammonia: ethanol: water, is calculated as 1.9:4.0:487:389. Finally, the resultant Fe_3_O_4_@SiO_2_ nanoparticle with core–shell structure was separated and obtained with a magnet and washed by ethanol at least 6 times.

#### 3.1.3. Fe_3_O_4_@SiO_2_@RF Fabrication

In a typical fabrication procedure, 100 mg of Fe_3_O_4_@SiO_2_ nanoparticles, resorcinol (0.55 g), hexamethylenetetramine (0.35 g), Pluronic F127 (1.00 g) and 1,3,5-tremethylbenzene (TMB) were mixed together in 18 mL DI water with vigorous magnetic stirring under room temperature for 120 min. The above homogenous mixture was then poured into an oven for 12 h at 100 °C. Finally, the orange-red nanoparticles were separated by filtration, washed by water or ethanol several times and freeze-dried for 24 h; Fe_3_O_4_@SiO_2_@RF with mesoporous shell was obtained.

#### 3.1.4. Yolk–Shell Fe_3_O_4_@hmC

Then, the resultant orange-red deposit of Fe_3_O_4_@SiO_2_@RF nanospheres was carbonized at 400 °C for 3 h with a heating rate at 2 °C/min under a pure nitrogen atmosphere, followed by further carbonization procedure at 800 °C for 3 h. Then, the Fe_3_O_4_@SiO_2_@C was successfully prepared. The solid silica shell was removed under 0.1 NaOH at 60 °C for 1 h. The final products were washed 3 times of water. The obtained yolk–shell Fe_3_O_4_@hmC was dispersed in water.

### 3.2. In Vivo Anti-Tumor Evaluation of Fe_3_O_4_@hmC

#### 3.2.1. Subcutaneous Tumor Model Construction

All animal experiments obtained approval from the Use Committee and Institutional Animal Care in Xiamen University. All animal studies were conducted with relevant guidelines (Ethics Approval: No. XMULAC20180037). Female BALB/c mice, 6–8 weeks old, were brought from Charles River Labs. Then, after 2 days feeding, BALB/c mice were given subcutaneous injection of 1 × 10^6^ 4T1 cells in 50 μL PBS at the lower limbs in right side. After 7 days of injection, tumor size grew into 80–100 mm^3^, the following MRI, photothermal imaging and tumor ablation, experiments could be performed. Tumor volumes were calculated by the standard formula: length × width^2^ × 0.5.

#### 3.2.2. In Vivo Tumor Eradication Efficiency

BALB/c mice with subcutaneous 4T1 breast tumor were evenly assigned into 4 groups and were given 2 intravenous injections at day 1 and day 7 during the whole tumor eradication procedure, for 14 days. Accordingly, various treatments (*n* = 4 per group): (1) PBS, (2) DOX, (3) Fe_3_O_4_@hmC-DOX (7 mg kg^−1^), (4) Fe_3_O_4_@hmC-DOX (7 mg kg^−1^) + 808 nm NIR laser irradiation (1.0 W/cm^2^, 5 min) at 12 h after intravenous injection. The tumor sizes were carefully detected by a conventional digital caliper. All tumor volumes and body weights in each group were recorded every 2 days. After 16 days treatment, all tumors and organs were dissected, and photos were taken of the tumors for intuitive observation of tumor size.

#### 3.2.3. Histopathological Evaluation

Tumors of (1) PBS, (2) DOX, (3) Fe_3_O_4_@hmC-DOX (7 mg kg^−1^), (4) Fe_3_O_4_@hmC-DOX (7 mg kg^−1^) + 808 nm NIR laser irradiation for 5 days treatment were resected and cut into tissue sections 10 μm thick. Then, H&E and TUNNEL staining were carried out. Meanwhile, the major organs, heart, spleen, liver, lung and kidney after (1) PBS, (2) DOX, (3) Fe_3_O_4_@hmC-DOX (7 mg kg^−1^), (4) Fe_3_O_4_@hmC-DOX (7 mg kg^−1^) + 808 nm NIR laser irradiation treatments at 16 days were also performed by H&E staining for biosafety evaluation.

## 4. Conclusions

In this work, we built a novel Fe_3_O_4_@ mesoporous carbon nanoparticle with a unique yolk–shell nanostructure for the first time. The hollowed cavity can be used as a nanocarrier for drug delivery; the Fe-based core can be applied for MRI contrast agent; more importantly, the carbon-based shell had effective photothermal conversion capability for hyperthermia-induced cellular cytotoxicity. The hollowed cavity of this carbon-based nanoparticle presented remarkably high membrane adsorption compared with conventional mesoporous carbon nanocomponents with core–shell nanostructure. Meanwhile, little cell viability was detected after 808 nm laser irradiation after incubation with Fe_3_O_4_@hmC-DOX. We found more effective tumor accumulation of Fe_3_O_4_@hmC in comparison with Fe_3_O_4_@SiO_2_@C in vivo, and anti-tumor studies demonstrated that our synergistic therapy of the chemo–photothermal effect exhibited total tumor ablation with low side effects. Our carbon-based yolk–shell nanocarrier holds potential for clinical applications in complete eradication of malignant tumors.

## Data Availability

Not applicable.

## Supplementary Materials

The following supporting information can be downloaded at: https://www.mdpi.com/article/10.3390/ijms23031623/s1.
