# Supplementary material for: A Novel Yolk–Shell Fe3O4@ Mesoporous Carbon Nanoparticle as an Effective Tumor-Targeting Nanocarrier for Improvement of Chemotherapy and Photothermal Therapy
Source: Int J Mol Sci. 2022 Jan 30;23(3):1623. doi: 10.3390/ijms23031623 (PMC8835829; doi:10.3390/ijms23031623)
Supplement: Supplementary file 1 [file ijms-23-01623-s001.zip › ijms-1564285-supplementary.pdf]

International Journal of *Molecular Sciences*

MDPI

Article

A Novel Yolk–Shell Fe_3O_4@ Mesoporous Carbon Nanoparticle as an Effective Tumor-Targeting Nanocarrier for Improvement of Chemotherapy and Photothermal Therapy

Haina Tian [1,2,†], Ruifeng Zhang [2,3,†], Jiaqi Li [2,3], Cailin Huang [2,3], Wen Sun [4], Zhenqing Hou [1,*] and Peiyuan Wang [2,*]

1 Research Center of Biomedical Engineering of Xiamen, Key Laboratory of Biomedical Engineering of Fujian Province, Department of Biomaterials, College of Materials, Xiamen University, Xiamen 361005, China; hainatian@163.com (H.T.)

2 Key Laboratory of Design and Assembly of Functional Nanostructures, Fujian Institute of Research on the Structure of Matter, Chinese Academy of Sciences, Fuzhou 350002, China; zhangruifeng@mail.ustc.edu.cn (R.Z.); lijq7@mail.ustc.edu.cn (J.L.); Hhhayu@163.com (C.H.)

3 School of Rare Earths, University of Science and Technology of China, Hefei 230026, China

4 State Key Laboratory of Fine Chemicals, Dalian University of Technology, 2 Linggong Road, Hi-tech Zone, Dalian 116024, China; sunwen@dlut.edu.cn (W. S.)

* Correspondence: wangpeiyuan@fjirsm.ac.cn (P.W.); houzhenqing@xmu.edu.cn (Z.H.)

† These authors contributed equally to this work.

Part A: Supplementary Experimental Section

Materials and characterization.

Materials. Anhydrous $FeCl_3$, trisodium citrate, sodium acetate, tetraethyl orthosilicate (TEOS), Resorcinol, formaldehyde, hexamethylenetetramine, ethanol, ethylene glycol, concentrated ammonia solution (28 wt %) are of analytical grade (Shanghai Chemical Corp.).Hexadecyltrimethylammonium bromide (CTAB) and dopamine hydrochloride were purchased from Sigma-Aldrich. NaOH, cyclohexane and NH_4NO_3 were obtained from Shanghai Chemical Co., Ltd. Ammonia aqueous solution (28 wt %), tetraethyl orthosilicate (TEOS), (3-Aminopropyl) triethoxysilane (APTES), bis[3-(triethoxysily)propyl] tetrasulfide (BTES), triethanolamine (TEA), decahydronaphthalene (98 %) were purchased from Aladdin Industrial Inc. All chemicals were used as received without further purification

Characterization. Transmission electron microscopy (TEM) measurements were carried out on a JEM 2100F microscope (Japan) operated at 200 kV. LEO1530VP SEM (Germany), a Bruker Multimode 8, high-resolution transmission electron microscope operating at 200 kV. SEM measurement was analyzed using Nanoscope V multimode atomic force microscope. The samples were first dispersed in ethanol and then collected by using copper grids covered with carbon films for measurements. UV–vis–NIR absorption spectra were measured on a Shimadz spectrophotometer (UV-3150) (Japan) with wavelength range of 300-1200 nm, unless otherwise specified, all spectra were collected under identical experimental conditions.

In vitro cellular targeting and cell viability of Fe_3O_4 @ vhmC

1. Cell viability

All cell viability experiments were studied in 96-well plates. Cytotoxicity of Fe_3O_4@hmC-DOX was tested *via* CCK-8 assay. Briefly, the primary 4T1 isolated through enzymatic digestion were seeded into plate at 5×10^3/well in 100 μL of 1640 (10% FBS, 100 units/mL of penicillin and 100 μg/mL of streptomycin), and incubated for 24 h. Then, Fe_3O_4@hmC with various concentration was added followed by 12 h incubation. Lately, 10 μL CCK-8 was added to the cells, and after 2 h incubation, the absorbance of each well

at wavelength of 450 nm was measured using a microplate reader. Data were presented as mean ± SD (n = 3). Meanwhile, PBS, DOX, Fe_3O_4@hmC-DOX with 1064 nm laser irradiation (0.5 W/cm^2, 5 min) was performed under the same procedure.

2. CLSM images of cellular uptake.

The cellular uptake of Fe_3O_4@hmC was observed and imaged by confocal laser scanning microscopy (CLSM). Briefly, 4T1 cells were seeded into 6-well plates at a density of 1×10^5/well with 1 mL of 1640 (10% FBS, 100 units/mL of penicillin and 100 μg/mL of streptomycin) media and incubated for 24 h. After the treatment with Fe_3O_4@hmC at a final concentration of 50 μg/mL, the 4T1 cells were incubated for different hours. Furthermore, Fe_3O_4@hmC and Fe_3O_4 @ SiO_2 @ C treated 4T1 cells for 8 h were also studied. Then, all the samples were washed with PBS for three times and DAPI (1 μg /mL in PBS) was used to stain nuclei for 30 min prior to being observed under CLSM.

3. Live/Dead cell staining and cell apoptosis analysis

Both calcein-AM/PI assay and Annexin V-FITC/PI apoptosis assays were also performed to evaluate the *in vitro* antitumor efficiency and mechanism. 4T1 cells seeded into 1×10^5/well with 1 mL of 1640 media (10% FBS, 100 units/mL of penicillin and 100 μg/mL of streptomycin) in 6-well plates for 24 h then the cells were treated by incubation of different formulations for 8 h. Then, PBS, DOX, Fe_3O_4@hmC-DOX, Fe_3O_4@hmC-DOX + laser treatment group was staining by calcein-AM/PI assay, the resultant cells were stained with calcein-AM and PI and imaged by CLSM. For the Annexin V-FITC/PI apoptosis assay, PBS, DOX, Fe_3O_4@hmC-DOX, Fe_3O_4@hmC-DOX + laser treatment group cells were stained by Annexin V-FITC and PI, and the percentage of apoptosis was analyzed by a flow cytometer.

Part B: Supplementary Figure Section

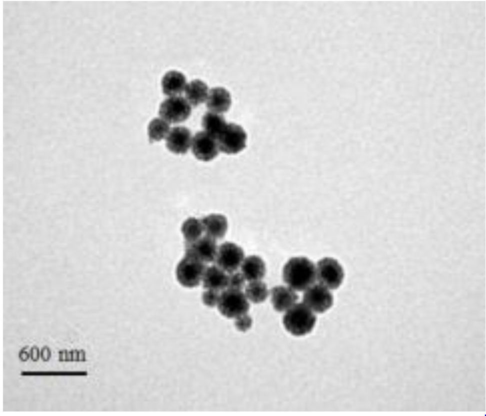

Figure S1. TEM image of Fe_3O_4@SiO_2@RF.

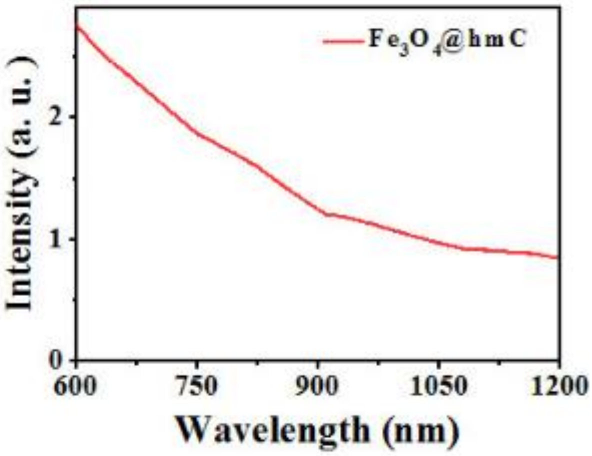

Figure S2. UV-vis-NIR absorbance spectra of Fe_3O_4@hmC.

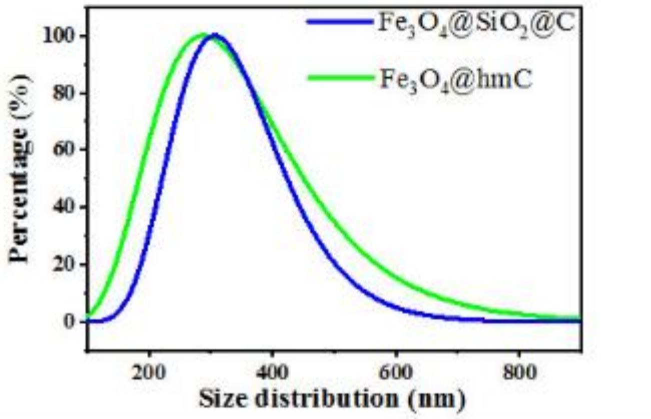

Figure S3. DLS analysis of Fe_3O_4@hmC and Fe_3O_4@SiO_2@C. (Fe_3O_4@SiO_2@C: ~306 nm and Fe_3O_4@hmC: ~294 nm).

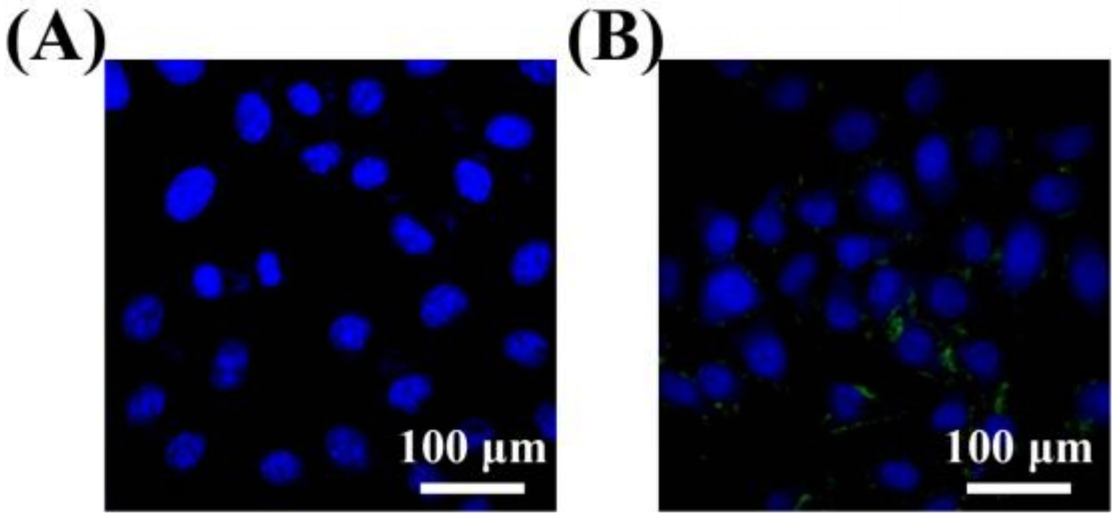

Figure S4. CLSM images of 4T1 cells treated with (A) Fe_3O_4@SiO_2@C and (B) Fe_3O_4@vhmC for 0.25 h incubation.

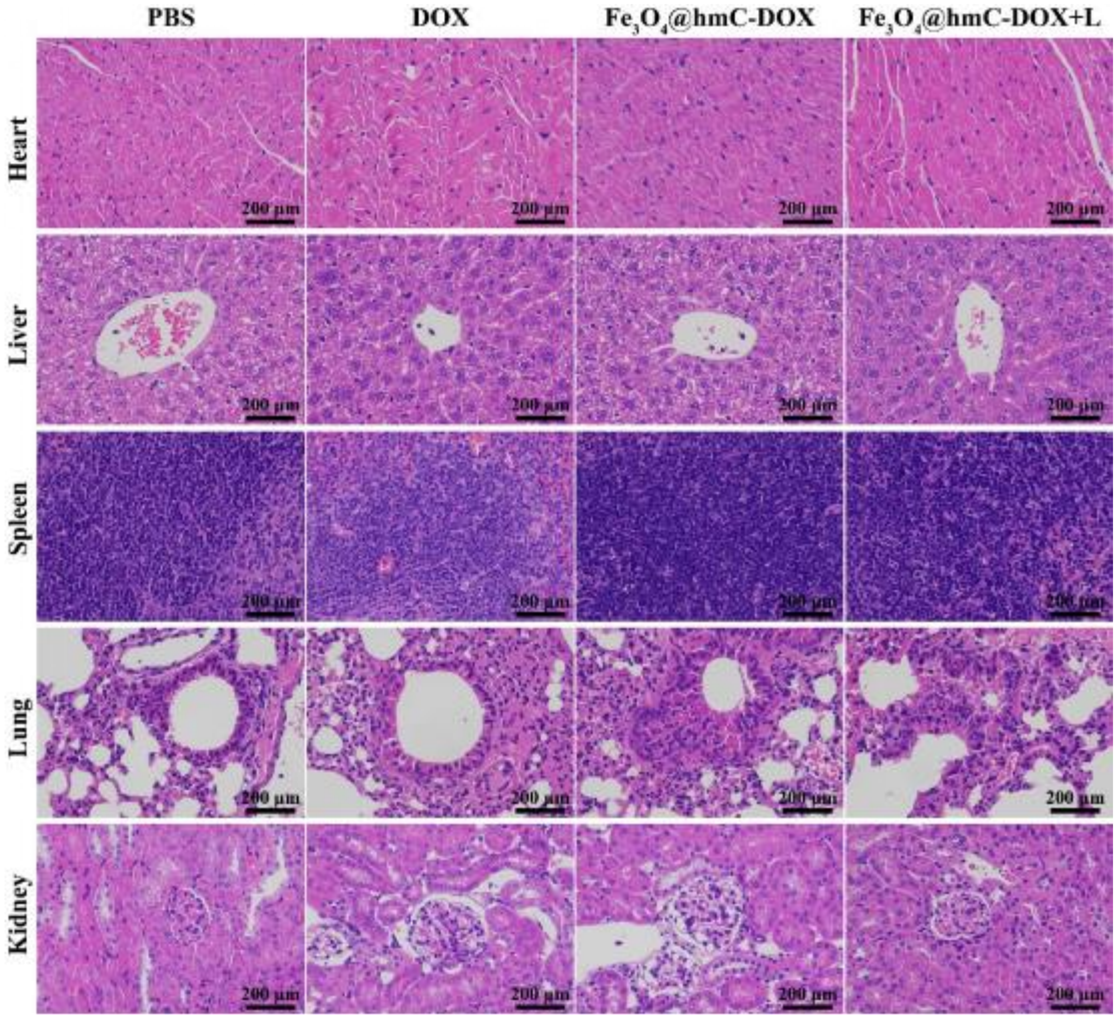

Figure S5. H&E staining of major organs after 14 days with different treatments.
